# The Difference in Electromyographic Activity While Wearing a Medical Mask in Women with and without Temporomandibular Disorders

**DOI:** 10.3390/ijerph192315559

**Published:** 2022-11-23

**Authors:** Michał Ginszt, Grzegorz Zieliński, Jacek Szkutnik, Marcin Wójcicki, Michał Baszczowski, Monika Litko-Rola, Diana Zielińska, Ingrid Różyło-Kalinowska

**Affiliations:** 1 Department of Rehabilitation and Physiotherapy, Medical University of Lublin, 20-093 Lublin, Poland; 2 Department of Sports Medicine, Medical University of Lublin, 20-093 Lublin, Poland; 3 Independent Unit of Functional Masticatory Disorders, Medical University of Lublin, 20-093 Lublin, Poland; 4 Interdisciplinary Scientific Group of Sports Medicine, Department of Sports Medicine, Medical University of Lublin, 20-093 Lublin, Poland; 5 Department of Dental and Maxillofacial Radiodiagnostics, Medical University of Lublin, 20-093 Lublin, Poland

**Keywords:** COVID-19, SARS-CoV-2, TMDs, surface electromyography, masticatory muscles, medical mask

## Abstract

Wearing a medical mask influences resting activity of the temporalis anterior and masseter muscles in healthy young women. However, no studies link medical mask-wearing with masticatory muscle activity in patients with temporomandibular disorders (TMDs). Therefore, this study aims to compare electromyographic patterns while wearing a medical mask between women with and without temporomandibular disorders. Based on the Research Diagnostic Criteria for Temporomandibular Disorders, 115 adult women qualified for the study. Participants were divided into the following two groups: diagnosed TMDs (*n* = 55; mean age: 23.5 ± 2.3 years) and healthy women (*n* = 60; mean age: 23.7 ± 2.6 years). Examinations of the resting and functional electromyographic activity of the temporalis anterior (TA), superficial masseter (MM), anterior bellies of the digastric muscle (DA), and the middle part of the sternocleidomastoid muscle (SCM) were carried out using the BioEMG III™. Both groups showed statistically significant decreases in resting masticatory muscle activity during medical mask examination compared to no mask measurement. The significant differences in no mask measurement between both groups were noted regarding resting masticatory activity, clenching in the intercuspal position, and clenching on dental cotton rollers. During medical mask examination, women with TMDs showed differences in resting masticatory activity and clenching on dental cotton rollers compared to the healthy group. In all analyzed variables, both groups showed similar electromyographic patterns in the maximum mouth opening measurement during medical mask and no mask examination. A medical mask influences the resting bioelectric activity of the masticatory muscles in women with temporomandibular disorders and healthy women. We observed differences and some similarities in resting and functional electromyographic patterns within masticatory and neck muscles in both groups during medical mask and no mask examination.

## 1. Introduction

The coronavirus disease 2019 (COVID-19) pandemic has influenced the typical way of life regarding medical care and daily functioning. General control strategies (e.g., social distancing, disinfection, and personal protection) were proposed and widely used to protect the population’s health during the COVID-19 pandemic. Preventive recommendations for COVID-19 include vaccination, physical distancing, and personal protective equipment, of which the most used are medical masks [1]. Medical masks are recommended to reduce the spreading of COVID-19 by lowing transmissibility per contact [2,3]. Moreover, medical masks protect the wearer from infection [4].

On the other hand, wearing medical masks can provoke adverse health effects. Besides feeling discomfort [5], mask wearers report headaches [6,7], breathing difficulties [8], and can experience dermatological problems [9]. The irritation of nerves and other soft tissue in the neck and head region caused by the medical mask straps also contributes to headaches [6].

As we previously reported, wearing a medical mask influences the resting activity of the temporalis anterior and masseter muscles in healthy young women [10]. However, our research did not analyze subjects with stomatognathic dysfunctions, such as temporomandibular disorders (TMDs). TMDs affect the temporomandibular joint, masticatory muscles, and surrounding tissues. These disorders are characterized by pain and disturbed muscle activity within the stomatognathic system [11]. Moreover, TMDs are related to alternations in the bioelectric patterns of masticatory muscles [12,13]. However, there is a lack of research on the effect of medical masks on masticatory muscle activity in patients with TMDs. Therefore, the present study aimed to compare electromyographic patterns between women with and without temporomandibular disorders while wearing medical masks. Moreover, we compare the electromyographic activity between no mask and medical mask conditions in both groups. Following the previous studies, the presented research hypothesizes that medical masks influence the bioelectric patterns of the masticatory and neck muscle activity in both groups. As TMDs affect muscle activity, the electromyographic patterns in both groups may differ under the medical mask condition. To the best of our knowledge, this is the first report that investigates differences in electromyographic patterns during medical mask-wearing in TMD patients.

## 2. Materials and Methods

### 2.1. Study Population

The study was carried out according to the recommendations of the Declaration of Helsinki and with the consent of the Bioethical Commission of the Medical University of Lublin (approval number: KE-0254/81/2021). The research was performed between October 2021 and March 2022 at the Independent Unit of Functional Masticatory Disorders, Medical University of Lublin, Poland. All participants were informed about the study’s aim and gave written consent.

Based on the Research Diagnostic Criteria for Temporomandibular Disorders [14], 115 female participants qualified for the research. Women were divided into the following two groups: diagnosed TMDs (*n* = 55; mean age: 23.5 ± 2.3 years) and the healthy group (*n* = 60; mean age: 23.7 ± 2.6 years). A skilled dentist diagnosed pain-related TMDs (myofascial pain within masticatory muscles). Patients with temporomandibular joint (TMJ) disorders and diseases, other muscle disorders (e.g., contracture, myositis and myospasm), and coronoid hyperplasia were excluded from the research. Moreover, the following exclusion criteria were used in the presented study: the occurrence of headaches and cervical spine pain within the month preceding the measurement; head and neck trauma within the last six months before the examination; Angle’s Class II and III; lack of four support zones and lack of more than four teeth in dental arches; periodontal diseases; open bite; orthodontic treatment; possession of dental prostheses; botulinum toxin treatment; neurological diseases; pregnancy.

After RDC/TMD examination, the ultrasound measurement was performed using an M-Turbo device (15–16 MHz linear transducer; 6 cm scan depth; SonoSite Inc, Bothell, WA, USA) to assess the TMJ structures and confirm the manual examination.

### 2.2. Research Protocol

**Figure 1:**
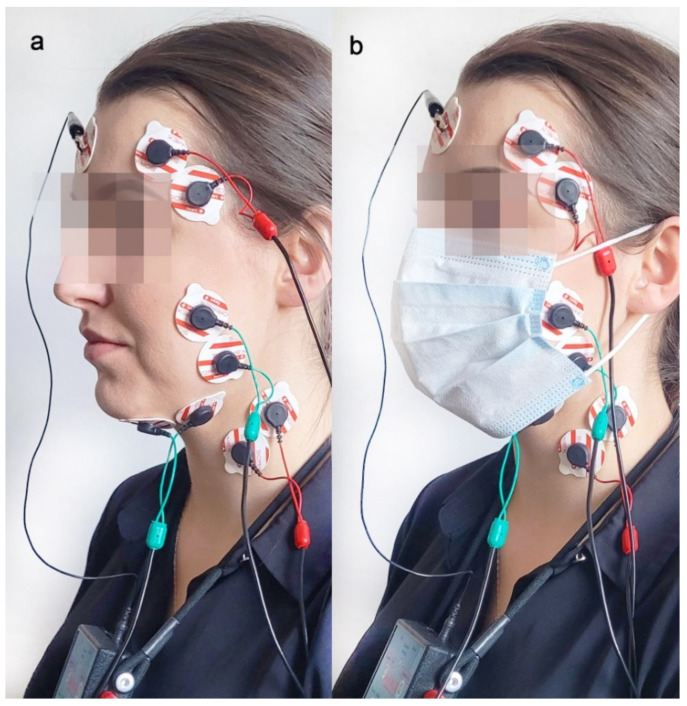
Electrode placement during two measurements: without (**a**) and with face mask (**b**).

The presented research consisted of the following two phases: (1) medical mask measurement; (2) no mask measurement. The initial measurement was chosen randomly. Study subjects completed four masticatory tasks with and without a certified 3-layer medical facemask (Type II 50PSC, 000-994, Abeba GmbH, St. Ingbert, Germany) with a 5 min break between phases. The face mask covered the participant’s mouth and nose (Figure 1). The placement of the medical mask did not provoke pain during the examination.

### 2.3. Electromyographic Examination

Measurements of the resting and functional electromyographic activity of the temporalis muscle (anterior part—TA), masseter muscle (superficial part—MM), digastric muscle (anterior bellies—DA), and sternocleidomastoid muscle (the middle part—SCM) were carried out using an 8-channel device for surface electromyography (BioEMG III™, BioResearch Associates, Inc., Milwaukee, WI, USA). The bioelectric activity was recorded during the following four masticatory activities: in the rest position of the mandible (10 s), during teeth clenching in the intercuspal contact area (3 times for 3 s each, with 2 s break), during teeth clenching on dental rollers (3 times for 3 s each, with 2 s break) and during active mouth opening (3 times for 3 s each, with 2 s break). The mean values of the three records of each activity were used for statistical calculations.

The electromyographic measurements were performed between 9 am and 11 am to reduce the influence of the daily bioelectric variability in muscles on the electromyographic patterns. The participants sat on a dental chair, with their head on the headrest and their torso perpendicular to the ground. The subject’s skin was cleansed with a 90% ethanol solution to lower skin impedance. The same physiotherapist placed surface electrodes on the participants (Ag/AgCl, 30 mm diameter, 16 mm conductive surface, SORIMEX, Toruń, Poland). The sEMG electrodes were placed following SENIAM standards [15]. The edges of the electrodes adhered to each other to standardize the distance between conductive surfaces, with the reference electrode placed on the frontal bone, as presented in Figure 1. The sEMG electrode placement has been described by us in line with a previous report [10].

### 2.4. sEMG Signal Processing and Statistical Calculations

**Figure 2:**
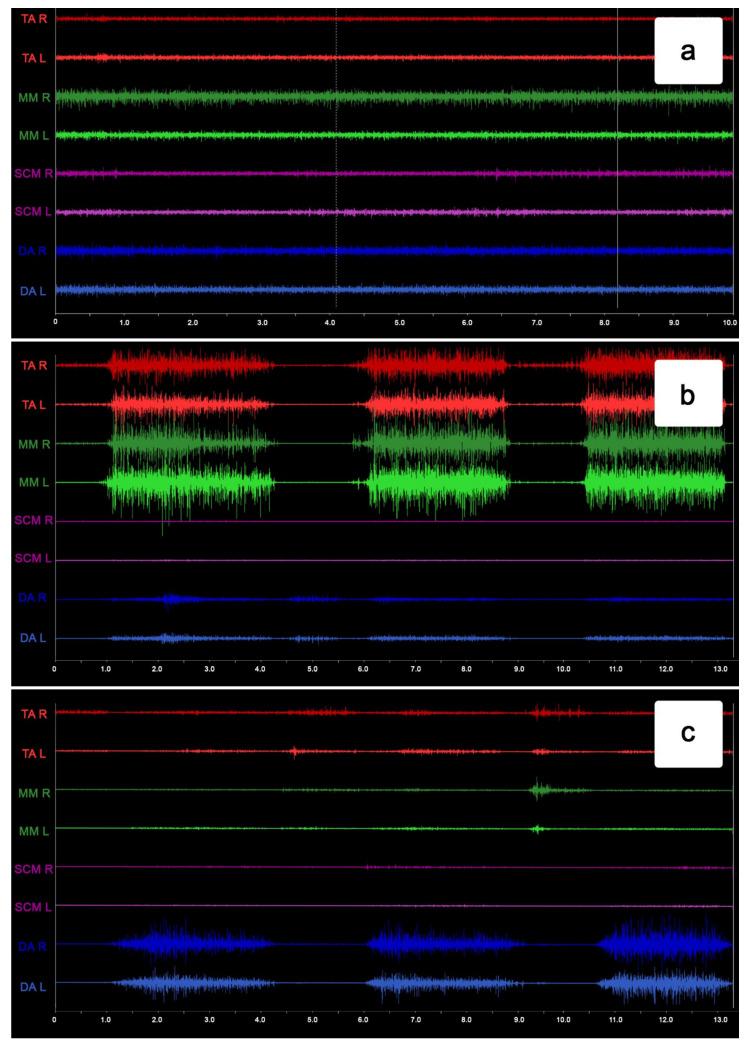
Example of the surface electromyography traces during resting activity (**a**), maximum voluntary clenching (**b**), and maximum mouth opening (**c**).

The STROBE inventory was used to check the research quality [16]. The repeatability of the electromyographic measurements was performed by duplicate sEMG records on ten subjects [10]. Microvolt values were amplified and reduced by 40 dB using the Noise Buster filtering. Impedance tests were administered to all participants before and after each measurement using a BioPAK system (BioResearch Associates, Inc., Milwaukee, WI, USA). Moreover, all the electromyographic signals were confirmed visually before RMS processing. Bioelectric signal processing based on the root mean square (RMS) formula was used to obtain the average electromyographic results (Figure 2).

The Functional Clenching Indices (FCI) and Functional Opening Indices (FOI) were used to normalize the mean electromyographic activity. FCI and FOI indices were calculated from the average RMS values, according to Ginszt and Zieliński’s study protocol [17]. The indices mentioned above were calculated from RMS potentials during teeth clenching (CL), maximum mouth opening (MMO), and resting activity (REST) using the following formulas:

FCI for TA right-sided (FCI_TA-R_) = CL_TA-R_/REST_TA-R_ (1)

FCI for TA left-sided (FCI_TA-L_) = CL_TA-L_/REST_TA-L_ (2)

FCI for TA both-sided (FCI_TA total_) = (CL_TA-R_ + CL_TA-L_)/(REST_TA-R_ + REST_TA-L_) (3)

FCI for MM right-sided (FCI_MM-R_) = CL_MM-R_/REST_MM-R_ (4)

FCI for MM left-sided (FCI_MM-L_) = CL_MM-L_/REST_MM-L_ (5)

FCI for MM both-sided (FCI_MM total_) = (CL_MM-R_ + CL_MM-L_)/(REST_MM-R_ + REST_MM-L_) (6)

FCI for SCM right-sided (FCI_SCM-R_) = CL_SCM-R_/REST_SCM-R_ (7)

FCI for SCM left-sided (FCI_SCM-L_) = CL_SCM-L_/REST_SCM-L_ (8)

FCI for SCM both-sided (FCI_SCM total_) = (CL_SCM-R_ + CL_SCM-L_)/(REST_SCM-R_ + REST_SCM-L_) (9)

FCI for DA right-sided (FCI_DA-R_) = CL_DA-R_/REST_DA-R_ (10)

FCI for DA left-sided (FCI_DA-L_) = CL_DA-L_/REST_DA-L_ (11)

FCI for DA both-sided (FCI_DA total_) = (CL_DA-R_ + CL_DA-L_)/(REST_DA-R_ + REST_DA-L_) (12)

FOI for TA right-sided (FOI_TA-R_) = MMO_TA-R_/REST_TA-R_ (13)

FOI for TA left-sided (FOI_TA-L_) = MMO_TA-L_/REST_TA-L_ (14)

FOI for TA both-sided (FOI_TA total_) = (MMO_TA-R_ + MMO_TA-L_)/(REST_TA-R_ + REST_TA-L_) (15)

FOI for MM right-sided (FOI_MM-R_) = MMO_MM-R_/REST_MM-R_ (16)

FOI for MM left-sided (FOI_MM-L_) = MMO_MM-L_/REST_MM-L_ (17)

FOI for MM both-sided (FOI_MM total_) = (MMO_MM-R_ + MMO_MM-L_)/(REST_MM-R_ + REST_MM-L_) (18)

FOI for SCM right-sided (FOI_SCM-R_) = MMO_SCM-R_/REST_SCM-R_ (19)

FOI for SCM left-sided (FOI_SCM-L_) = MMO_SCM-L_/REST_SCM-L_ (20)

FOI for SCM both-sided (FOI_SCM total_) = (MMO_SCM-R_ + MMO_SCM-L_)/(REST_SCM-R_ + REST_SCM-L_) (21)

FOI for DA right-sided (FOI_DA-R_) = MMO_DA-R_/REST_DA-R_ (22)

FOI for DA left-sided (FOI_DA-L_) = MMO_DA-L_/REST_DA-L_ (23)

FOI for DA both-sided (FOI_DA total_) = (MMO_DA-R_ + MMO_DA-L_)/(REST_DA-R_ + REST_DA-L_) (24)

Calculations were performed using IBM SPSS Statistics 13.3 (TIBCO Software Inc., Palo Alto, CA, USA). The normal distribution of the data was verified with the Kolmogorov–Smirnov test (with Lilliefors correction) and the Shapiro–Wilk test. The results did not have a normal distribution. Therefore, a non-parametric test was used. To compare groups, the Mann–Whitney U-test was used. Effect sizes were calculated for the *t*-test using the Cohen d method and interpreted as large (0.8), medium (0.5), and small (0.2) effect sizes [18,19]. Statistical significance was set at *p* ≤ 0.05.

## 3. Results

### 3.1. Group Overview

There were no significant differences in age (*p* = 0.86), weight (*p* = 0.91), height (*p* = 0.69), body mass index (*p* = 0.86), or maximum active mouth opening range of motion (*p* = 0.06) between the TMD group and healthy women. However, the TMD group presented lower values for maximum active mouth opening than healthy participants (48.73 vs. 45.76; *p* = 0.06), as shown in Table 1.

### 3.2. Electromyographic Results

We observed a significant decrease in left (3.41 µV vs. 2.57 µV; *p* = 0.02; ES = 0.24) and mean (3.28 µV vs. 2.59 µV; *p* = 0.03; ES = 0.23) TA electromyographic activity for the medical mask condition compared to the no mask measurement in the resting mandibular position. Significant decreases in resting RMS results were also found for the medical mask condition compared to the no mask measurement concerning the right MM (2.45 µV vs. 1.89 µV; *p* = 0.02; ES = 0.25). In terms of remaining RMS values, the differences between measurements did not reach the significance level (*p* > 0.05) (Table 2).

We found a significant decrease in resting potentials in the right MM (2.00 µV vs. 1.74 µV; *p* = 0.04; ES = 0.25), left MM (2.20 µV vs. 1.86 µV; *p* = 0.01; ES = 0.24) and mean MM values (2.10 µV vs. 1.80 µV; *p* = 0.02; ES = 0.26) during medical mask examination compared to the no mask measurement. In terms of other RMS values, the differences did not reach the significance level (*p* > 0.05) (Table 3).

We observed significant differences between the mask and no mask examinations in the functional clenching indices for FCI TA (right, left, and total), FCI SCM (right, left, and total), and FCI DA (right, left, and total) during clenching in the intercuspal position in TMD patients. All FCI values for the temporalis anterior and digastric muscles were significantly lower during medical mask examination than in no mask examination. The opposite tendency was demonstrated within the SCM, where the medical mask caused a significant increase in functional indices. In terms of other functional indices, the differences did not reach the significance level (*p* > 0.05) (Table 4).

As presented in Table 5, significant differences between no mask and medical mask examination were observed in the functional clenching indices for FCI TA (right, left, and total), FCI SCM (right, left, and total), and FCI DA (right, left, and total) during clenching in the intercuspal position in the control group. All FCI values for the temporalis anterior and digastric muscles were significantly lower in the medical mask examination than in no mask examination. The opposite tendency was demonstrated within the SCM, where medical masks caused a significant increase in the functional indices. In terms of other functional indices, the differences did not reach the significance level (*p* > 0.05) (Table 5).

We observed significant differences between TMD patients and controls in the functional clenching indices within the SCM (right, left, and total) and digastric muscle (left and total) during intercuspal teeth clenching during no mask examination. All FCI values were significantly lower in the TMD group than in healthy subjects. Similar results were obtained during clenching on dental rollers within the TA (left), SCM (right, left, and total), and digastric muscle (left and total), where patients with TMDs presented lower values of FCI indices. Only the FCI for the MM (right) showed significantly higher values in the TMD group than in the controls.

During maximum mouth opening, the controls presented higher values of the FOI SCM (right) than TMD subjects. The differences between the two groups did not reach the significance level in the remaining functional indices (*p* > 0.05) (Table 6).

During intercuspal teeth clenching, the controls presented significantly higher values of the FCI for the temporalis anterior (total) than TMD subjects in medical mask examination. During dental roller clenching, significant differences were found in the MM (right and total), SCM (right, left, and total), and digastric muscle (right, left, and total), where the patients with TMDs presented lower values of FCI indices.

During maximum mouth opening, the controls presented higher values of functional opening indices within the masseter (right, total), SCM (total), and digastric muscle (right) than the TMD subjects. The remaining results did not reach the assumed significance level (*p* > 0.05) (Table 7).

## 4. Discussion

The current scientific reports indicate that a medical mask reduces COVID-19 transmissibility. Moreover, the available epidemiological data confirm that wearing medical masks is the most effective method to reduce the spread of the coronavirus [20]. However, extended mask-wearing could provoke relevant side effects and medical consequences. Mask wearers can be at risk of mask-induced exhaustion syndrome (MIES), with signs and symptoms such as an increase in breathing dead space volume [21], an increase in breathing resistance [22], an increase in blood carbon dioxide [23], decrease in blood oxygen saturation [24], shortness of breath and difficulty breathing [25], and headache [26]. Moreover, wearing a face mask influences the resting activity of the masticatory muscles [10]. Thus, the side effects of face masks are clinically relevant, and a risk-benefit analysis is essential for minimizing medical masks’ side effects [27].

As we previously reported, wearing a face mask influences masticatory muscle activity in healthy women [10]. However, there is a lack of studies that investigate the influence of using medical masks on the electromyographic patterns in subjects with TMDs. TMDs affect masticatory muscles and influence electromyographic patterns within the stomatognathic system [11,12]. Therefore, the present study aimed to compare electromyographic patterns between women with and without temporomandibular disorders during medical mask conditions. Moreover, we compared the electromyographic activity between no mask and medical mask conditions in both groups. To the best of our knowledge, this is the first report that investigates electromyographic patterns in TMD patients during medical mask-wearing. We hypothesized that using a medical mask significantly influences electromyographic patterns in women with TMDs and healthy women.

As we assumed, the electromyographic patterns were significantly different under the medical mask condition in comparison to the no mask condition in both TMD and healthy groups. We observed statistically significant differences between no mask and medical mask examination in the functional clenching indices within the temporalis anterior, sternocleidomastoid, and digastric muscles in healthy women and women with TMDs. The results indicate that FCI values for the temporalis anterior and digastric muscles were significantly lower during medical mask examination than in no mask examination. Surprisingly, the opposite tendency was demonstrated within the SCM, where the medical mask caused a significant increase in functional indices. The differences mentioned above may be due to the different functions of the examined muscles during teeth clenching. Medical masks have been associated with decreased functional activity of the masticatory muscles. These associations have been observed in both jaw-closing and jaw-opening muscles. In the SCM muscle, the mask caused an increase in functional activity. It may be related to the increased stabilization of the cervical spine muscles in response to the decreased functional activity in the area of the masticatory muscles. The SCM muscle has been reported as an essential muscle that provides head and neck stability during mastication [28]. On the other hand, it is one of the most significant muscles that influences TMDs and is referred to as a pain muscle in the stomatognathic systems [29]. Moreover, elevated muscle activity of the SCM was observed in TMD patients [30]. Thus, changes in activity within masticatory and cervical spine muscles under medical mask conditions appear to resemble electromyographic patterns in patients with TMDs. During clenching in the intercuspal position, healthy women presented significantly higher functional activity values for the temporalis anterior than TMD subjects during medical mask examination. However, the difference between the groups in the temporalis anterior was not observed in the no mask condition. Therefore, it can be assumed that the medical masks significantly affected the activity of the anterior temporalis muscles in the group of TMD patients, changing the electromyographic patterns in this group compared to the controls. Differences in response to the mask between groups indicate some similarities in electromyographic patterns in both groups. However, in terms of women with TMDs, the differences under the influence of the medical mask seem to be more significant. It may be due to altered muscle activity in the TMD patient group, which is even more visible when a medical mask is worn. Therefore, further research on wearing a medical mask by TMD patients is fundamental in terms of understanding the disturbances in masticatory muscle activity. Moreover, it is necessary to examine the exact mechanism of SCM muscle activation under the influence of a medical mask and check what medical consequences it will cause in the stabilization of the head and the stomatognathic system.

Finally, we should stress that our results are limited by the short-term follow-up used in this study. Moreover, the research sample consists of young female participants. Thus, future research may include a long-term observation period and the male population. In addition, future electromyographic studies may use modern semi-dry electrodes, which facilitate bioelectrical signal pathways and significantly reduce electrode–skin impedance [31].

## 5. Conclusions

To summarize, we observed changes in electromyographic patterns due to medical mask-wearing in TMD patients and healthy women. Despite the many similarities in the electromyographic changes in both groups, more significant changes due to wearing a medical mask were observed in patients with muscular dysfunctions within the stomatognathic system. Several possible explanations for this observation (e.g., the difference in reaction to the pressure of the mask straps, ventilation disorders, biomechanical changes within the stomatognathic system, and psychological aspects) should be considered in future research.

## Figures and Tables

**Table 1:** General characteristics of participants include age, weight, height, body mass index (BMI), and maximum mouth opening (MMO) range of motion.

	Healthy Group *n* = 60		TMD Group *n* = 55		Z	*p*
	M	SD	M	SD		
Age (years)	23.73	2.61	23.52	2.32	−0.17	0.86
Weight (kg)	60.19	8.70	61.68	11.19	0.11	0.91
Height (cm)	168.10	6.39	168.39	6.37	0.39	0.69
BMI (kg/m^2^)	21.28	2.83	21.70	3.46	0.32	0.75
MMO (mm)	48.73	6.08	45.76	7.98	−1.92	0.06

TMDs—temporomandibular disorders; Z—Mann–Whitney U test; M—mean; SD—standard deviation; BMI—body mass index; MMO—maximum active mouth opening.

**Table 2:** The comparison of the root mean square (RMS) electromyographic activity between no mask and medical mask conditions in the TMD group.

		No Mask Measurement TMD Group (*n* = 55)		Medical Mask Measurement TMD Group (*n* = 55)		Z	*p/*ES
		M (µV)	SD (µV)	M (µV)	SD (µV)		
Rest	TA R	3.15	2.35	2.62	2.27	1.38	0.17
	TA L	3.41	2.74	2.57	2.06	2.28	0.02 */0.24
	TA Mean	3.28	2.18	2.59	1.93	2.14	0.03 */0.23
	MM R	2.45	1.50	1.89	0.84	2.34	0.02 */0.25
	MM L	2.29	1.39	1.88	0.98	1.63	0.10
	MM Mean	2.37	1.32	1.89	0.76	1.86	0.06
	SCM R	1.35	0.53	1.18	0.35	1.57	0.12
	SCM L	1.45	0.62	1.32	0.49	1.22	0.22
	SCM Mean	1.40	0.53	1.25	0.37	1.44	0.15
	DA R	2.08	0.94	2.25	1.63	0.12	0.91
	DA L	2.01	0.83	2.12	1.43	0.76	0.45
	DA Mean	2.05	0.83	2.19	1.42	0.56	0.57
	TA R	127.79	76.96	116.41	66.71	0.83	0.40
Clenching in the intercuspal position	TA L	123.71	67.36	109.34	67.94	1.26	0.21
	TA Mean	125.75	67.25	112.88	64.05	1.04	0.30
	MM R	137.97	107.10	115.60	98.81	1.46	0.15
	MM L	132.51	101.91	111.82	94.83	1.31	0.19
	MM Mean	135.24	101.98	113.71	94.59	1.44	0.15
	SCM R	8.89	7.55	7.87	7.26	1.29	0.20
	SCM L	8.57	8.05	10.95	26.47	0.41	0.68
	SCM Mean	8.73	7.57	9.41	15.03	0.89	0.37
	DA R	19.30	12.05	16.95	11.48	1.47	0.14
	DA L	17.40	13.73	15.53	12.51	0.95	0.34
	DA Mean	18.35	12.24	16.24	11.40	1.43	0.15
Clenching on dental cotton rollers	TA R	137.44	160.23	110.52	51.47	0.57	0.57
	TA L	113.10	54.86	104.95	56.65	0.96	0.34
	TA Mean	125.27	91.17	107.73	51.45	0.74	0.46
	MM R	173.14	161.56	144.30	92.06	0.89	0.37
	MM L	148.03	88.07	133.65	81.21	0.90	0.37
	MM Mean	160.58	110.38	138.97	84.20	1.03	0.30
	SCM R	10.79	7.54	9.45	6.70	1.31	0.19
	SCM L	10.21	7.23	9.19	6.80	0.85	0.40
	SCM Mean	10.50	7.08	9.32	6.39	1.03	0.30
	DA R	21.31	11.37	19.29	10.09	1.00	0.32
	DA L	18.84	11.05	15.88	8.79	1.50	0.13
	DA Mean	20.07	10.55	17.59	8.63	1.35	0.18
Maximum mouth opening	TA R	6.89	3.83	6.85	3.75	0.07	0.94
	TA L	6.87	3.91	6.72	3.77	0.09	0.93
	TA Mean	6.88	3.50	6.78	3.28	0.07	0.94
	MM R	7.65	5.67	7.67	6.36	0.29	0.77
	MM L	7.98	7.82	7.73	7.58	0.44	0.66
	MM Mean	7.81	6.60	7.70	6.87	0.36	0.72
	SCM R	8.90	7.46	8.63	6.65	−0.01	0.99
	SCM L	8.00	5.60	8.10	5.22	−0.27	0.79
	SCM Mean	8.45	6.34	8.36	5.67	−0.12	0.90
	DA R	72.65	44.04	77.29	41.11	−0.84	0.40
	DA L	77.40	48.17	77.88	42.32	−0.54	0.59
	DA Mean	75.03	44.16	77.59	39.00	−0.75	0.45

TMDs—temporomandibular disorders; Z—Mann–Whitney U test; ES—effect size; M—mean; SD—standard deviation; TA—the temporalis anterior; MM—the superficial part of the masseter muscle; SCM—the middle part of the sternocleidomastoid muscle; DA—the anterior belly of the digastric muscle; R—right side; L—left side; μV—microvolt; *—significant difference.

**Table 3:** The comparison of the root mean square (RMS) electromyographic activity between no mask and medical mask conditions in healthy women.

		No Mask Measurement Healthy Group (*n* = 60)		Medical Mask Measurement Healthy Group (*n* = 60)		Z	*p/*ES
		M (µV)	SD (µV)	M (µV)	SD (µV)		
Rest	TA R	2.60	1.93	2.10	1.29	1.50	0.13
	TA L	2.57	1.50	2.17	1.46	1.90	0.06
	TA Mean	2.58	1.48	2.14	1.17	1.78	0.08
	MM R	2.00	1.09	1.74	1.09	2.08	0.04 */0.25
	MM L	2.20	1.27	1.86	1.49	2.45	0.01 */0.24
	MM Mean	2.10	1.05	1.80	1.19	2.43	0.02 */0.26
	SCM R	1.17	0.32	1.12	0.32	1.10	0.27
	SCM L	1.32	0.42	1.23	0.40	1.29	0.20
	SCM Mean	1.24	0.33	1.17	0.30	0.99	0.32
	DA R	1.91	1.05	1.92	1.12	0.33	0.74
	DA L	1.87	1.07	1.83	1.05	0.25	0.81
	DA Mean	1.89	1.04	1.87	1.06	0.23	0.82
	TA R	147.70	89.48	130.58	81.23	1.08	0.28
Clenching in the intercuspal position	TA L	140.25	74.83	124.06	70.26	1.06	0.29
	TA Mean	143.97	79.13	127.32	73.97	1.06	0.29
	MM R	150.77	99.07	125.60	91.15	1.67	0.10
	MM L	146.23	102.84	124.72	92.69	1.33	0.19
	MM Mean	148.50	97.97	125.16	89.43	1.48	0.14
	SCM R	10.81	8.14	8.52	5.83	1.66	0.10
	SCM L	10.68	8.17	8.80	6.24	1.27	0.20
	SCM Mean	10.75	7.78	8.66	5.54	1.41	0.16
	DA R	22.06	14.68	19.62	13.46	0.98	0.33
	DA L	24.38	19.84	19.53	15.04	1.50	0.13
	DA Mean	23.22	15.48	19.58	13.28	1.33	0.19
Clenching on dental cotton rollers	TA R	132.67	76.61	133.72	76.23	−0.25	0.80
	TA L	126.42	67.38	127.60	67.55	−0.15	0.88
	TA Mean	129.54	70.06	130.66	69.39	−0.21	0.83
	MM R	167.28	91.75	160.27	79.63	0.15	0.88
	MM L	163.60	95.43	155.42	85.41	0.15	0.88
	MM Mean	165.44	90.53	157.85	78.08	0.22	0.83
	SCM R	12.71	7.62	13.76	14.97	0.68	0.50
	SCM L	12.17	7.12	11.81	7.94	0.45	0.66
	SCM Mean	12.44	6.96	12.78	9.56	0.48	0.63
	DA R	22.89	11.46	22.32	10.55	0.23	0.82
	DA L	24.73	14.44	23.14	14.58	0.88	0.38
	DA Mean	23.81	11.98	22.73	11.71	0.55	0.58
Maximum mouth opening	TA R	7.44	4.50	10.05	19.98	−0.09	0.93
	TA L	7.11	4.74	7.04	4.97	0.57	0.57
	TA Mean	7.28	4.23	8.54	10.75	0.09	0.93
	MM R	9.65	9.05	10.80	10.73	−0.31	0.76
	MM L	8.82	6.49	9.91	7.76	−0.16	0.87
	MM Mean	9.23	7.46	10.35	8.91	−0.27	0.79
	SCM R	9.49	6.74	11.63	11.27	−0.77	0.44
	SCM L	9.53	8.03	10.91	10.99	−0.58	0.56
	SCM Mean	9.51	7.07	11.27	10.69	−0.55	0.58
	DA R	80.12	40.79	84.24	42.03	−0.52	0.60
	DA L	78.91	40.53	86.01	42.14	−0.86	0.39
	DA Mean	79.51	38.66	85.13	39.75	−0.66	0.51

Z—Mann–Whitney U test; ES—effect size; M—mean; SD—standard deviation; TA—the temporalis anterior; MM—the superficial part of the masseter muscle; SCM—the middle part of the sternocleidomastoid muscle; DA—the anterior belly of the digastric muscle; R—right side; L—left side; μV—microvolt; *—significant difference.

**Table 4:** The comparison of the functional indices between no mask and medical mask measurements in the TMD group.

		No Mask Measurement (*n* = 55)		Medical Mask Measurement (*n* = 55)		Z	*p/*ES
		M	SD	M	SD		
Clenching in the intercuspal position	FCI TA R	63.55	57.45	10.72	7.49	7.26	0.00 */0.80
	FCI TA L	61.62	62.80	9.83	8.23	7.26	0.00 */0.80
	FCI TA Total	59.04	55.23	10.18	7.42	7.38	0.00 */0.82
	FCI MM R	78.60	99.26	66.24	57.52	−0.07	0.94
	FCI MM L	73.09	71.39	68.81	67.72	0.56	0.57
	FCI MM Total	73.80	81.34	63.83	58.01	0.43	0.67
	FCI SCM R	7.09	6.89	77.58	95.38	−7.84	0.00 */0.87
	FCI SCM L	6.37	6.53	74.43	81.53	−7.76	0.00 */0.86
	FCI SCM Total	6.68	6.59	73.72	84.80	−7.94	0.00 */0.88
	FCI DA R	10.72	7.49	6.95	6.25	3.61	0.00 */0.40
	FCI DA L	9.83	8.23	9.39	26.94	3.23	0.00 */0.36
	FCI DA Total	10.18	7.42	8.10	15.55	3.71	0.00 */0.41
Clenching on dental cotton rollers	FCI TA R	61.14	53.59	61.01	43.82	−0.51	0.61
	FCI TA L	55.01	53.46	64.98	59.10	−1.17	0.24
	FCI TA Total	55.30	49.76	59.27	45.17	−1.04	0.30
	FCI MM R	104.85	172.97	94.97	83.12	−1.05	0.29
	FCI MM L	80.95	59.48	87.65	70.28	−0.47	0.64
	FCI MM Total	87.07	85.73	87.64	72.04	−0.78	0.44
	FCI SCM R	8.42	6.01	8.47	6.12	0.05	0.96
	FCI SCM L	7.61	5.84	7.42	5.83	0.25	0.81
	FCI SCM Total	7.93	5.76	7.76	5.44	0.19	0.85
	FCI DA R	11.67	6.87	10.59	7.47	1.11	0.27
	FCI DA L	10.50	6.62	9.38	6.33	0.87	0.38
	FCI DA Total	10.98	6.36	9.83	6.42	0.97	0.33
Maximum mouth opening	FOI TA R	3.21	2.24	3.68	2.52	−1.17	0.24
	FOI TA L	2.93	2.36	4.01	3.58	−1.53	0.13
	FOI TA Total	2.92	2.02	3.69	2.66	−1.62	0.11
	FOI MM R	4.08	3.74	5.01	4.95	−1.37	0.17
	FOI MM L	4.66	5.14	5.14	4.84	−1.18	0.24
	FOI MM Total	4.21	4.20	4.88	4.60	−1.19	0.23
	FOI SCM R	7.03	5.84	7.71	6.16	−1.24	0.21
	FOI SCM L	6.05	4.45	6.65	4.62	−0.94	0.35
	FOI SCM Total	6.43	4.78	6.99	4.74	−1.14	0.25
	FOI DA R	40.82	32.53	42.13	26.81	−0.68	0.50
	FOI DA L	44.46	40.74	45.17	29.97	−0.81	0.42
	FOI DA Total	3.21	2.24	3.68	2.52	−1.17	0.24

TMDs—temporomandibular disorders; Z—Mann–Whitney U test; ES—effect size; M—mean; SD—standard deviation; FCI—Functional Clenching Index; FOI—Functional Opening Index; TA—the temporalis anterior; MM—the superficial part of the masseter muscle; SCM—the middle part of the sternocleidomastoid muscle; DA—the anterior belly of the digastric muscle; R—right side; L—left side; *—significant difference.

**Table 5:** The comparison of the functional indices between no mask and medical mask measurements in the healthy group.

		No Mask Measurement (*n* = 60)		Medical Mask Measurement (*n* = 60)		Z	*p/*ES
		M	SD	M	SD		
Clenching in the intercuspal position	FCI TA R	74.62	55.54	13.62	10.31	8.16	0.00 */0.86
	FCI TA L	70.54	49.09	15.08	14.57	7.66	0.00 */0.81
	FCI TA Total	68.24	45.44	14.31	11.18	8.15	0.00 */0.86
	FCI MM R	97.52	93.64	84.33	82.60	0.76	0.45
	FCI MM L	88.94	82.61	83.88	75.35	0.30	0.77
	FCI MM Total	90.76	83.24	78.40	70.87	0.71	0.48
	FCI SCM R	10.03	9.17	94.88	89.62	−8.35	0.00 */0.88
	FCI SCM L	8.97	8.57	95.90	97.09	−7.77	0.00 */0.82
	FCI SCM Total	9.40	8.26	92.75	85.48	−8.13	0.00 */0.86
	FCI DA R	13.62	10.31	8.06	5.68	3.34	0.00 */0.35
	FCI DA L	15.08	14.57	7.73	6.05	4.21	0.00 */0.45
	FCI DA Total	14.31	11.18	7.77	5.26	3.89	0.00 */0.41
Clenching on dental cotton rollers	FCI TA R	65.02	46.62	80.23	67.41	−1.38	0.17
	FCI TA L	62.86	39.76	80.67	61.44	−1.14	0.26
	FCI TA Total	60.42	37.55	74.22	52.07	−1.32	0.19
	FCI MM R	102.15	80.10	113.17	75.37	−1.19	0.23
	FCI MM L	97.89	81.92	113.40	98.29	−1.04	0.30
	FCI MM Total	97.68	76.96	110.06	77.66	−1.20	0.23
	FCI SCM R	11.75	8.75	13.06	13.87	−0.32	0.75
	FCI SCM L	10.18	7.55	10.45	7.99	0.00	1.00
	FCI SCM Total	10.83	7.61	11.66	9.77	−0.12	0.90
	FCI DA R	14.22	8.03	14.71	9.38	0.10	0.92
	FCI DA L	15.24	10.24	14.77	10.79	0.62	0.53
	FCI DA Total	14.65	8.49	14.61	9.34	0.38	0.71
Maximum mouth opening	FOI TA R	3.79	2.88	7.19	20.51	−1.35	0.18
	FOI TA L	3.54	3.15	4.32	3.63	−1.63	0.10
	FOI TA Total	3.45	2.41	4.79	5.03	−1.65	0.10
	FOI MM R	6.43	9.19	8.27	12.39	−1.51	0.13
	FOI MM L	5.53	6.26	7.44	8.41	−1.86	0.06
	FOI MM Total	5.85	7.36	7.73	10.13	−1.56	0.12
	FOI SCM R	8.46	5.90	10.99	10.51	−0.93	0.35
	FOI SCM L	7.62	6.17	9.18	8.09	−1.24	0.21
	FOI SCM Total	7.93	5.81	9.87	8.50	−1.00	0.32
	FOI DA R	51.19	34.77	54.12	34.31	−0.72	0.47
	FOI DA L	51.64	34.77	56.64	36.13	−1.03	0.30
	FOI DA Total	51.17	33.80	55.03	34.43	−0.81	0.42

TMDs—temporomandibular disorders; Z—Mann–Whitney U test; ES—effect size; M—mean; SD—standard deviation; FCI—Functional Clenching Index; FOI—Functional Opening Index; TA—the temporalis anterior; MM—the superficial part of the masseter muscle; SCM—the middle part of the sternocleidomastoid muscle; DA—the anterior belly of the digastric muscle; R—right side; L—left side; *—significant difference.

**Table 6:** The comparison of the functional indices between the TMD group and healthy women during no mask examination.

		Healthy Group (*n* = 60)		TMD Group (*n* = 55)		Z	*p/*ES
		M	SD	M	SD		
Clenching in the intercuspal position	FCI TA R	74.62	55.54	63.55	57.45	−1.46	0.14
	FCI TA L	70.54	49.09	61.62	62.80	−1.84	0.07
	FCI TA Total	68.24	45.44	59.04	55.23	−1.85	0.06
	FCI MM R	97.52	93.64	78.60	99.26	−1.79	0.07
	FCI MM L	88.94	82.61	73.09	71.39	−1.30	0.19
	FCI MM Total	90.76	83.24	73.80	81.34	−1.65	0.10
	FCI SCM R	10.03	9.17	7.09	6.89	−2.57	0.01 */0.28
	FCI SCM L	8.97	8.57	6.37	6.53	−2.61	0.01 */0.28
	FCI SCM Total	9.40	8.26	6.68	6.59	−2.82	0.00 */0.31
	FCI DA R	13.62	10.31	10.72	7.49	−1.42	0.16
	FCI DA L	15.08	14.57	9.83	8.23	−2.76	0.01 */0.30
	FCI DA Total	14.31	11.18	10.18	7.42	−2.24	0.02 */0.24
Clenching on dental cotton rollers	FCI TA R	65.02	46.62	61.14	53.59	−1.00	0.32
	FCI TA L	62.86	39.76	55.01	53.46	−2.02	0.04 */0.22
	FCI TA Total	60.42	37.55	55.30	49.76	−1.63	0.10
	FCI MM R	102.15	80.10	104.85	172.97	−2.12	0.03 */0.23
	FCI MM L	97.89	81.92	80.95	59.48	−1.09	0.28
	FCI MM Total	97.68	76.96	87.07	85.73	−1.59	0.11
	FCI SCM R	11.75	8.75	8.42	6.01	−3.11	0.00 */0.34
	FCI SCM L	10.18	7.55	7.61	5.84	−2.83	0.00 */0.31
	FCI SCM Total	10.83	7.61	7.93	5.76	−3.19	0.00 */0.35
	FCI DA R	14.22	8.03	11.67	6.87	−1.87	0.06
	FCI DA L	15.24	10.24	10.50	6.62	−3.24	0.00 */0.35
	FCI DA Total	14.65	8.49	10.98	6.36	−2.72	0.01 */0.29
Maximum mouth opening	FOI TA R	3.79	2.88	3.21	2.24	−1.08	0.28
	FOI TA L	3.54	3.15	2.93	2.36	−0.87	0.39
	FOI TA Total	3.45	2.41	2.92	2.02	−1.11	0.27
	FOI MM R	6.43	9.19	4.08	3.74	−1.93	0.05
	FOI MM L	5.53	6.26	4.66	5.14	−0.83	0.40
	FOI MM Total	5.85	7.36	4.21	4.20	−1.50	0.13
	FOI SCM R	8.46	5.90	7.03	5.84	−2.16	0.03 */0.23
	FOI SCM L	7.62	6.17	6.05	4.45	−1.72	0.09
	FOI SCM Total	7.93	5.81	6.43	4.78	−1.95	0.05
	FOI DA R	51.19	34.77	40.82	32.53	−1.71	0.09
	FOI DA L	51.64	34.77	44.46	40.74	−1.37	0.17
	FOI DA Total	51.17	33.80	42.23	35.71	−1.58	0.11

TMDs—temporomandibular disorders; Z—Mann–Whitney U test; ES—effect size; M—mean; SD—standard deviation; FCI—Functional Clenching Index; FOI—Functional Opening Index; TA—the temporalis anterior; MM—the superficial part of the masseter muscle; SCM—the middle part of the sternocleidomastoid muscle; DA—the anterior belly of the digastric muscle; R—right side; L—left side; *—significant difference.

**Table 7:** The comparison of the functional indices between the TMD group and healthy women during medical mask examination.

		Healthy Group (*n* = 60)		TMD Group (*n* = 55)		Z	*p/*ES
		M	SD	M	SD		
Clenching in the intercuspal position	FCI TA R	13.62	10.31	10.72	7.49	−1.42	0.16
	FCI TA L	15.08	14.57	9.83	8.23	−2.76	0.01
	FCI TA Total	14.31	11.18	10.18	7.42	−2.24	0.02 */0.24
	FCI MM R	84.33	82.60	66.24	57.52	−1.25	0.21
	FCI MM L	83.88	75.35	68.81	67.72	−1.35	0.18
	FCI MM Total	78.40	70.87	63.83	58.01	−1.45	0.15
	FCI SCM R	94.88	89.62	77.58	95.38	−1.48	0.14
	FCI SCM L	95.90	97.09	74.43	81.53	−1.48	0.14
	FCI SCM Total	92.75	85.48	73.72	84.80	−1.42	0.15
	FCI DA R	8.06	5.68	6.95	6.25	−1.72	0.09
	FCI DA L	7.73	6.05	9.39	26.94	−1.80	0.07
	FCI DA Total	7.77	5.26	8.10	15.55	−1.83	0.07
Clenching on dental cotton rollers	FCI TA R	80.23	67.41	61.01	43.82	−1.78	0.08
	FCI TA L	80.67	61.44	64.98	59.10	−1.82	0.07
	FCI TA Total	74.22	52.07	59.27	45.17	−1.93	0.05
	FCI MM R	113.17	75.37	94.97	83.12	−2.01	0.04 */0.22
	FCI MM L	113.40	98.29	87.65	70.28	−1.88	0.06
	FCI MM Total	110.06	77.66	87.64	72.04	−2.06	0.04 */0.22
	FCI SCM R	13.06	13.87	8.47	6.12	−3.22	0.00 */0.35
	FCI SCM L	10.45	7.99	7.42	5.83	−3.13	0.00 */0.34
	FCI SCM Total	11.66	9.77	7.76	5.44	−3.42	0.00 */0.37
	FCI DA R	14.71	9.38	10.59	7.47	−2.63	0.01 */0.28
	FCI DA L	14.77	10.79	9.38	6.33	−3.32	0.00 */0.36
	FCI DA Total	14.61	9.34	9.83	6.42	−3.04	0.00 */0.33
Maximum mouth opening	FOI TA R	7.19	20.51	3.68	2.52	−1.06	0.29
	FOI TA L	4.32	3.63	4.01	3.58	−0.77	0.44
	FOI TA Total	4.79	5.03	3.69	2.66	−0.98	0.33
	FOI MM R	8.27	12.39	5.01	4.95	−2.21	0.03 */0.24
	FOI MM L	7.44	8.41	5.14	4.84	−1.88	0.06
	FOI MM Total	7.73	10.13	4.88	4.60	−2.11	0.04 */0.23
	FOI SCM R	10.99	10.51	7.71	6.16	−1.85	0.06
	FOI SCM L	9.18	8.09	6.65	4.62	−1.87	0.06
	FOI SCM Total	9.87	8.50	6.99	4.74	−2.04	0.04 */0.22
	FOI DA R	54.12	34.31	42.13	26.81	−1.99	0.05 */0.22
	FOI DA L	56.64	36.13	45.17	29.97	−1.68	0.09
	FOI DA Total	55.03	34.43	43.00	25.80	−1.83	0.07

TMDs—temporomandibular disorders; Z—Mann–Whitney U test; ES—effect size; M—mean; SD—standard deviation; FCI—Functional Clenching Index; FOI—Functional Opening Index; TA—the temporalis anterior; MM—the superficial part of the masseter muscle; SCM—the middle part of the sternocleidomastoid muscle; DA—the anterior belly of the digastric muscle; R—right side; L—left side; *—significant difference.

## Data Availability

The data presented in this study are available upon request from the corresponding author.
